# Motion-related artefacts in EEG predict neuronally plausible patterns of activation in fMRI data

**DOI:** 10.1016/j.neuroimage.2011.06.094

**Published:** 2012-01-02

**Authors:** Marije Jansen, Thomas P. White, Karen J. Mullinger, Elizabeth B. Liddle, Penny A. Gowland, Susan T. Francis, Richard Bowtell, Peter F. Liddle

**Affiliations:** a Division of Psychiatry, School of Community Health Sciences, University of Nottingham, UK; b Sir Peter Mansfield Magnetic Resonance Centre, School of Physics and Astronomy, University of Nottingham, Nottingham, UK

**Keywords:** EEG/fMRI, Artefacts, Motion, Attention, Network

## Abstract

The simultaneous acquisition and subsequent analysis of EEG and fMRI data is challenging owing to increased noise levels in the EEG data. A common method to integrate data from these two modalities is to use aspects of the EEG data, such as the amplitudes of event-related potentials (ERP) or oscillatory EEG activity, to predict fluctuations in the fMRI data. However, this relies on the acquisition of high quality datasets to ensure that only the correlates of neuronal activity are being studied. In this study, we investigate the effects of head-motion-related artefacts in the EEG signal on the predicted T2*-weighted signal variation. We apply our analyses to two independent datasets: 1) four participants were asked to move their feet in the scanner to generate small head movements, and 2) four participants performed an episodic memory task. We created T2*-weighted signal predictors from indicators of abrupt head motion using derivatives of the realignment parameters, from visually detected artefacts in the EEG as well as from three EEG frequency bands (theta, alpha and beta). In both datasets, we found little correlation between the T2*-weighted signal and EEG predictors that were not convolved with the canonical haemodynamic response function (cHRF). However, all convolved EEG predictors strongly correlated with the T2*-weighted signal variation in various regions including the bilateral superior temporal cortex, supplementary motor area, medial parietal cortex and cerebellum. The finding that movement onset spikes in the EEG predict T2*-weighted signal intensity only when the time course of movements is convolved with the cHRF, suggests that the correlated signal might reflect a BOLD response to neural activity associated with head movement. Furthermore, the observation that broad-spectral EEG spikes tend to occur at the same time as abrupt head movements, together with the finding that abrupt movements and EEG spikes show similar correlations with the T2*-weighted signal, indicates that the EEG spikes are produced by abrupt movement and that continuous regressors of EEG oscillations contain motion-related noise even after stringent correction of the EEG data. If not properly removed, these artefacts complicate the use of EEG data as a predictor of T2*-weighted signal variation.

## Highlights

► We studied the effects of movement artefacts during simultaneous EEG and fMRI. ► Movement artefacts are evident in different EEG frequency bands. ► EEG movement artefacts cause spurious correlations with the T2*-weighted signal. ► Results show the need to develop techniques to detect and remove movement artefacts.

## Introduction

The simultaneous acquisition of electroencephalography (EEG) and functional magnetic resonance imaging (fMRI) data provides the opportunity to study brain function at both high temporal and spatial resolution. Integration of EEG and T2*-weighted fMRI signals, however, is complex with a number of substantial challenges to overcome. One challenge arises from our limited understanding of the relationship between the T2*-weighted signal and underlying neuronal activity (Logothetis et al., 2001). Despite a growing literature suggesting differential correlation between T2*-weighted signals and EEG oscillatory activity in specific frequency bands (Mukamel et al., 2005), the optimal approach for integrating the two signals is yet to be formalised and is currently an area of extensive research (Kilner et al., 2005; Moeller et al., 2011; Ostwald et al., 2010). Secondly, there is a considerable technical challenge to produce EEG data of high enough quality to enable the integration of data from the two modalities, primarily on account of the degradation of EEG data collected in an MR environment.

There are two main sources of degradation of EEG data in the MR environment. First, EEG data is contaminated with gradient artefacts of large amplitude, originating from the temporal manipulation of magnetic field gradients required for the production of MR images (Allen et al., 2000). Second, pulse artefacts entrained to the cardiac pulse cycle, and believed to arise from associated movements of the head and electrodes within the B_0_ magnetic field (Yan et al., 2010), affect the EEG data in a manner which is not completely predictable. The unpredictability of this artefact arises from variations in the cardiac cycle and current deficiencies in the precise identification of the source of the artefact (Debener et al., 2008; Yan et al., 2010). The use of algorithms for artefact correction, often based on the formation and subtraction of average gradient- and pulse-artefact templates (e.g. Allen et al., 1998, 2000; Freyer et al., 2009), is a fundamental component of the pre-processing of EEG data acquired in an MR environment. The difficulties associated with removing the pulse artefact have also led to the development of a number of other methods, many of which draw on blind source separation techniques such as independent component analysis (ICA) and optimal basis sets (OBS; e.g. Debener et al., 2007; Freyer et al., 2009; Leclercq et al., 2009; Niazy et al., 2005). In addition, several techniques have been developed to improve artefact correction through improved acquisition methods (Debener et al., 2008; Mandelkow et al., 2006; Mullinger et al., 2008, 2011).

While head and electrode movements are believed to underlie the pulse artefact, it is clear that not all head or electrode movements are directly linked to the cardiac cycle. Head rotation in the strong static magnetic field of the scanner, not tightly related to cardiac phase, represents a third potential source of artefact and one that is less often considered. Small rotations of the head, whether pulse or non-pulse driven, cause the EEG electrodes and leads to move through the B_0_-field, potentially cutting lines of magnetic flux. This induces a voltage in the leads which results in the recording of an artefactual signal that is non-neuronal in origin. The amplitude of these voltages depends on the trajectory and speed of movement of the leads, as well as on the electrode positions and head shape.

Our observations suggest that head-movement artefacts are often characterised by their large amplitude, spike-like temporal profile and by their broad-spectral effects. Coordinated inter-dependent frequency band responses in scalp EEG have been reported during numerous information-processing tasks (for example, Demiralp et al., 2007; Griesmayr et al., 2010) and furthermore broad-band power suppression has been reported during visual working memory (Palva et al., 2010). It is nonetheless probable that the biophysical processes that underlie oscillations at different frequencies are at least partially dissociable (Başar et al., 2001; Kopell et al., 2010). Insofar as the cerebrally generated oscillations at different frequencies are generated by partially dissociable processes, it would be expected that the spectrum of the artefacts generated by head movements would have a more uniform broad band power spectrum than the signal generated directly by neural activity.

Head movements also have the secondary effect of altering the amplitude and temporal form of the gradient artefacts owing to changes in the position of the head and EEG leads relative to the MR gradient fields (Yan et al., 2009). The resulting variation in the gradient artefacts compromises the efficacy of the process of average-artefact subtraction. To overcome this problem, the average template is commonly formed from a sliding average. Recent attempts to improve gradient-artefact correction using the fMRI realignment parameters (Moosmann et al., 2009) illustrate a potential way in which information from MR data may assist in correcting artefacts in the EEG data. We note, however, that the use of realignment parameters, which consist of a single data point per brain volume, limits such correction to the detection of large, slow movements.

In summary, movements cause two types of artefacts: an interaction between the leads and the B_0_ field causing broad-spectral spikes, and a change in gradient-artefact morphology caused by changing the spatial orientation of leads relative to the MR gradients. The latter can be corrected to some extent by using adaptations of sliding-window, average artefact subtraction techniques, but the former is not always accounted for. The timing, speed and direction of head movements are unpredictable and therefore cause varying artefactual signals to be added to the EEG trace. These head movements may be linked to study design as participants may be more likely to move during particular stages of the experiment; for example, a button press following a stimulus may result in a consistent head nod. Since the extent of such movements varies between participants and over the duration of an fMRI experiment for a given participant, these artefacts can be difficult to identify and remove. This becomes a significant issue when considering the correlation of the T2*-weighted signal variation with modulation of a specific frequency band of EEG activity.

One commonly used technique for integrating simultaneously acquired EEG and fMRI data is to construct the time course of a particular frequency band of interest from the EEG data for the entire duration of the fMRI experiment. This time course is then convolved with the canonical haemodynamic response function (cHRF) and used in a general linear model (GLM) to identify regions of the brain where the T2*-weighted signal correlates with the predicted neuronal activity in that frequency band (Goldman et al., 2002; Laufs et al., 2003; Scheeringa et al., 2008). A variety of methods to reduce the effect of motion artefacts have been applied in this type of analysis. Goldman et al. (2002) visually inspected their EEG data and replaced artefactual periods, defined as showing minor motion or muscle artefacts, with interpolated values from neighbouring time points. Laufs et al. (2003) visually inspected data in order to detect EEG disturbance, as defined by “eye movements, gross motion and other artefacts”, as well as evidence that participants had fallen asleep. Based on these criteria, 5 out of 15 participants were excluded from further analysis in this study. In the remaining datasets, EEG amplitudes that exceeded three standard deviations from the mean were set to the respective standard deviation of the EEG time series. Scheeringa et al. (2008) transformed their theta frequency (2–9 Hz) time series to a time series of z-scores, and replaced any z-score greater than five with a zero.

Here, we investigate the effects of head-motion related artefacts in EEG data on the T2*-weighted signal correlates of such a continuous EEG regressor. We first address the hypothesis that large amplitude spikes in the frequency-specific EEG amplitude are observed at times of large head movement. We then investigate the T2*-weighted signal correlates of such artefacts by considering (i) direct correlates with these artefacts and (ii) correlates observed by convolving these spikes with a cHRF. We hypothesise that the onset of movement artefacts in the EEG data should coincide with movement artefacts in the MR data, causing direct T2*-weighted signal correlates of these artefacts around the perimeter of the brain and the cerebrospinal fluid. Adding a haemodynamic delay, i.e. by convolving the predictor of EEG artefacts with the cHRF, should provide a proxy for neural activity at the time of a head movement revealing a network of cortical regions associated with alerting, motor planning and motor execution processes.

We used two paradigms to study these hypotheses: one designed to elicit small head movements typical of those found in a standard fMRI task, but occurring at pre-set periods during scanning (foot movement task), and the second a typical EEG/fMRI experiment (episodic memory task) where head movements are less predictable. Since theta activity is modulated in memory tasks (e.g. Jensen and Tesche, 2002; Klimesch, 1999) such as the episodic memory task used in this study, we concentrate on studying the correlations of the theta EEG band with the T2*-weighted signal.

## Methods

### Data collection

EEG data were collected using an MR-compatible cap equipped with 31 Ag/AgCl electrodes positioned on the scalp according to the extended international 10–20 system (Braincap MR, Brain Products, Germany) with an additional electrooculography (EOG) electrode. An MR-plus Brain Amp with a bandwidth of 0.1–250 Hz and Brain Vision recorder (version 1.10) were used for data acquisition. The EEG data from both tasks were acquired at a 5 kHz sample rate, with FCz as the reference. The EEG sampling and imaging gradient waveforms were synchronised by driving the EEG amplifier's clock cycle using a 5 kHz signal derived from the 10 MHz reference signal from the MR scanner (Mandelkow et al., 2006). Cardiac and respiratory data were recorded using a vector cardiogram (VCG; Chia et al., 2000) and respiratory belt whose outputs were sampled at 500 Hz. R-peak markers derived from the VCG trace were temporally aligned with the EEG data using in-house software (Mullinger et al., 2008).

MRI data were collected using a 3 Tesla Philips Achieva MR scanner (Philips, The Netherlands) with whole body transmit coil and 8-channel receive head coil. Functional data were acquired using a multi-slice gradient-echo echo-planar imaging (GE-EPI) sequence. During the foot movement task, 250 volumes were acquired (TE = 40 ms, 4 mm slice thickness and 3 mm^2^ in-plane resolution, 32 descending axial slices in TR = 2.5 s). During the episodic memory task a dual-echo acquisition was performed with 330 dual-echo volumes acquired (TE = 20 and 48 ms, 4 mm slice thickness and 3 mm^2^ in-plane resolution, 40 descending axial slices in TR = 3 s). Multi-echo fMRI data acquisition was used for the episodic memory task since it has been shown to provide a more uniform sensitivity in detecting activation across brain regions with different T2* values (Gowland and Bowtell, 2007; Posse et al., 1999). Participants were instructed to minimise head movements while in the scanner. A magnetisation prepared rapid acquisition gradient echo (MPRAGE) sequence with 1 mm^3^ isotropic resolution, 256 × 256 × 160 matrix and SENSE factor 2 was additionally acquired to provide an anatomical image for each participant.

### Experimental paradigms

Studies were approved by the local-ethics committee and all participants gave informed written consent prior to participation in studies.

#### Foot movement task

Four participants (two males, 27 ± 3 years; mean ± standard deviation) performed a task designed to elicit small head movements at prescribed intervals. This task was designed to mimic movements often observed when participants are in longer fMRI sessions. Prior to scanning, participants were shown how to move their feet: a repeated dorsoflexion and plantarflexion of both ankles in tandem when presented with the ON-period visual cue (“wiggle”) and to remain still during the OFF-period (fixation cross). The block-design task included 16 cycles of a 5-second ON-period followed by a 30-second OFF-period. The visual cues (“wiggle” and fixation cross) were presented through goggles driven by fibre optics (Nordic Neurolab, Norway).

#### Episodic memory task

Four participants (three males, 31 ± 4 years) performed two runs of a 16-minute, event-related, visual episodic memory task (Gruber et al., 2008) and were instructed to keep their head still while doing so. Head movements during this task are expected to be typical of many other EEG/fMRI paradigms. Each run comprised a 6.5-minute learning phase, followed by a 30-second rest period and then a 9-minute retrieval test phase. During the learning phase, the participants viewed 40 pictures of objects and had to judge whether these were natural or artificial objects. The pictures were presented on a black background in either the upper or lower part of the screen for 1.2 s, followed by a button-press judgement for 1.5 s. Each trial finished with a rest period of pseudo-random duration of between 3 and 5 s. During the retrieval phase, the participants were presented with the same 40 pictures, intermixed with 20 new pictures. Pictures were presented for 2 s and following each presentation participants had to judge whether they had seen the object before, and in which location they had seen it. This was done by scrolling a tracker-ball (fORP, Cambridge Instruments, UK) to the object's previous location or, in the case of new objects, to the side of the screen. Again, a rest period of duration 3–5 s concluded the trial. Participants were instructed to attend to a central fixation cross during all inter-stimulus periods.

### Data analysis

#### EEG pre-processing

Initial data pre-processing was performed in BrainVision Analyzer 1.5 (Brain Products, Germany). We applied gradient artefact correction (Allen et al., 2000), a band-pass filter (0.5–40 Hz) and a notch-filter (50 Hz) to correct for the mains artefact. Data were subsequently down-sampled to 500 Hz and exported to EEGlab (http://sccn.ucsd.edu/eeglab/), where cardio-ballistic artefact correction was carried out with the plug-in FMRIB 1.2 that applies optimal basis sets (OBS; Niazy et al., 2005). In OBS, 3 principal components were used to create a set of basis functions describing the cardiac pulse artefact. These were then fitted to and removed from the EEG data. We did not apply any further correction to the foot movement dataset in order to maintain the artefacts caused by the movements. For the episodic memory dataset we wanted a signal that was comparable to that used previously by other researchers. We therefore used ICA without dimensionality reduction to select components that reflected: (i) residual artefacts related to gradient modulation; (ii) the cardiac pulse (by selecting the 3 components that contributed most to the temporal variation around the epoch of the heartbeat R-peak); (iii) eye movements; (iv) bad channels as identified by the component's topography. For the four participants in the episodic memory task dataset, we removed 8, 5, 9 and 5 of the 31 components, respectively.

#### fMRI pre-processing

Pre-processing and analysis of fMRI data was done using SPM8. Datasets were initially realigned to the first volume and slice-time corrected with the first slice used as a reference. For the episodic memory data, a weighted mean of the two echoes was then calculated to produce one dataset per volume (Gowland and Bowtell, 2007; Posse et al., 1999). Data for both tasks were corrected for physiological artefacts using retrospective image correction (RETROICOR; Glover et al., 2000). Data were then co-registered to the standard MNI template and spatially smoothed with a 5 mm full width at half maximum kernel.

#### EEG/fMRI integration

**Fig. 1:**
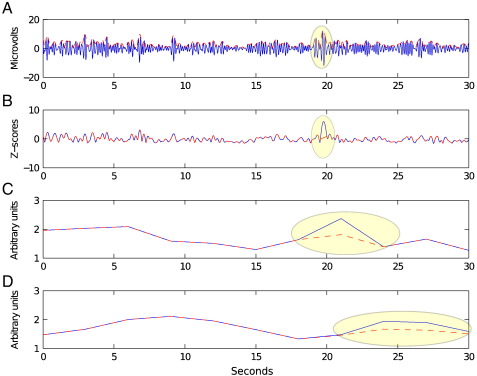
Method used to derive the theta predictor. A) EEG theta (4–8 Hz) signal fluctuations over 30 s (blue, solid line) and associated Hilbert envelope (red, dashed line), recorded at Cz. B) z-scored Hilbert envelope during the same 30 s, averaged over four fronto-central channels (Cz, FCz, FC1 and FC2). Blue solid line is the original time course, and the red dashed line the time course after applying a z threshold of 4 and interpolating to fill in the rejected values. Highlighted in yellow: time points where the signal exceeded the threshold. C) Fluctuations of the Hilbert envelope as pictured in B, without convolution with the cHRF, and D) after convolution with the canonical HRF.

The relationship between movement-related EEG artefacts and the T2*-weighted signal intensity changes was assessed using four different analyses:

(1) A simple box-car analysis based on the ON and OFF-periods of the *foot movement* task was initially used to study the T2*-weighted signal correlates of foot movements as well as the effects of consequential head movements. A GLM design matrix was formed from the ON and OFF-periods of the paradigm convolved with the cHRF. Realignment parameters were also included as covariates of no interest. To study artefactual effects of head movements on the MR signal, we repeated this GLM analysis without convolution of the paradigm with the cHRF.
(2) Head movements, as assessed from the realignment parameters, were related to T2*-weighted signal changes in datasets from both tasks. Realignment parameters give a volume-specific measure of head movement. The six realignment parameters per participant (x,y,z translations and 3 rotations: pitch, yaw and roll) were converted to z-scores by comparison with the mean and variance of each realignment parameter time course. We then extracted the first derivative of each time course (of which units are volumes), and squared the result. Then these six time courses were summed and z-scored again. We identified volumes within each dataset where this z-scored, sum of time courses exceeded 1, to create a single time series of delta functions indicative of supra-threshold movements. This threshold was chosen since it provided a good indication when head movements had taken place during the foot movement task. This time series – referred to as the abrupt movement regressor hereafter – was then used in a GLM to assess how the variation of the T2*-weighted signal correlated with abrupt movements. Two separate GLM design matrices were formed: the first using the abrupt movement regressor, permitting the assessment of MR artefacts produced instantaneously at times of movement; the second of the abrupt movement regressor convolved with the cHRF to assess neuronal activity related to the movement. For the episodic memory dataset, additional regressors consisting of convolved predictors of presentation of relevant task stimuli and non-convolved realignment parameters were also included in each GLM design matrix; for the foot movement task, non-convolved realignment parameters were included in each GLM design matrix but the regressor for convolved task stimuli was excluded on account of its colinearity with the abrupt movement regressors. Orthogonality of all included regressors was verified.
(3) Temporal fluctuations in EEG theta amplitude were extracted and used to predict concurrent T2*-weighted signal variation in datasets from *both* tasks. We hypothesised that continuous ‘theta regressors’ contain movement-related noise artefacts even after stringent cleaning (as described in the section Data collection). We tested the predictive effects of an EEG frequency band regressor, with and without convolution with the cHRF. For both datasets, estimates of theta, alpha and beta fluctuations were obtained from the EEG data following appropriate band-pass filtering (theta: 4–8 Hz, alpha: 8–12 Hz, beta: 12–30 Hz). The average Hilbert envelope of the signal fluctuations was taken over channels FC1, FC2, FCz and Cz in order to focus on fronto-central theta (Onton et al., 2005; Scheeringa et al., 2008), and this was converted to a z-score time course. Segments during which the data exceeded a z-score of 4, which is a slightly stricter criterion than that used by Scheeringa et al. (2008), were discarded and replaced by linear interpolation between segments (see Fig. 1). The theta regressor was entered into a GLM as an effect of interest including additional ‘nuisance’ regressors consisting of convolved task-related predictors and realignment parameters. This procedure was repeated for the alpha and beta bands.
(4) We visually inspected the EEG data of the *episodic memory* task to identify data segments in which significant EEG disturbance was evident. Two experienced EEG observers (TPW and MJ) inspected the EEG data independently, and a series of delta functions formed indicating volumes during which EEG disturbance was identified by both observers. We used these ‘EEG artefact regressors’ with and without convolution with the cHRF in two separate GLMs.

In summary, analyses 1,2 and 3 were applied to the foot movement data sets, and analyses 2,3 and 4 were applied to the episodic memory data sets. All analyses were performed twice: once with and once without convolution of the relevant regressor with the cHRF. Multiple-participant, fixed-effects analyses were performed to assess the consistency of results across participants, and to facilitate the comparison of results between the different analysis methods. For these fixed effects analyses, voxels were considered significant at a family-wise error corrected threshold of p < 0.05. The small sample size of the current sample precluded meaningful random-effects analysis.

## Results

**Fig. 2:**
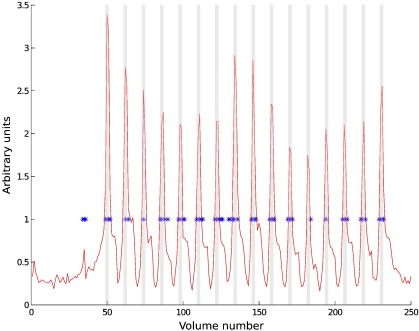
The theta regressor (not convolved with the cHRF) the foot movement task (red line) for a representative participant. FMRI Volume numbers during which this participant moved are indicated by the abrupt movement regressor, as derived from realignment parameters (blue asterisks). Grey bars indicate the period during which the participant was cued to move their feet.

Fig. 2 compares the times at which head movements were automatically detected from the realignment parameters to the measured EEG theta fluctuations, for one representative participant performing the foot movement task. Clear spikes in EEG theta signal related to foot movements are visible and head movements were also detected at times of cued foot movements. Head movements did not exceed 2 mm at any point in time. Furthermore, in the episodic memory task, spikes were found to occur in the alpha and beta regressors concurrently with spikes in the theta regressor. Consequently there were strong correlations between these regressors (all r > 0.8, all p < 0.0001), suggesting the same artefactual source of spikes in the different frequency bands (see Supplemental Fig. 1).

**Fig. 3:**
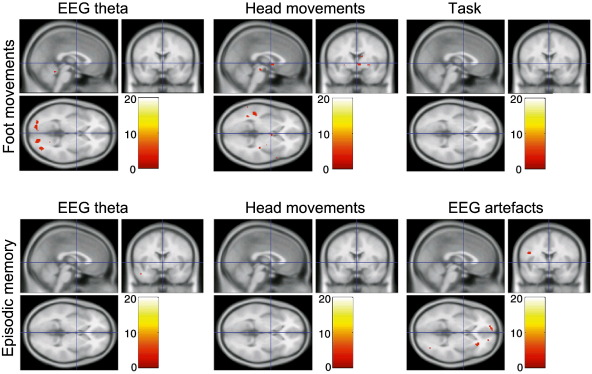
Fixed effects analysis results for the foot movement (n = 4; top row) and episodic memory (n = 4; bottom row) task for GLM regressors that were not convolved with the cHRF. T2*-weighted signal correlates of amplitude variation in the EEG theta band are shown in the left column, of abrupt head movements in the middle column, of the foot movement task instructions in the upper right corner and of visually detected artefacts in the EEG in the lower right corner. Fixed effects analysis, threshold at p < 0.05 family-wise error (FWE) corrected. See Table 1 for details of cluster locations and t-scores.

There was little evidence of correlation of the T2*-weighted signal with regressors that were not formed by convolution with the cHRF. This was true for both the foot-movement and episodic memory tasks. Clusters that did survive thresholding were typically of small size and low t-value (see Table 1A and Fig. 3).

**Fig. 4:**
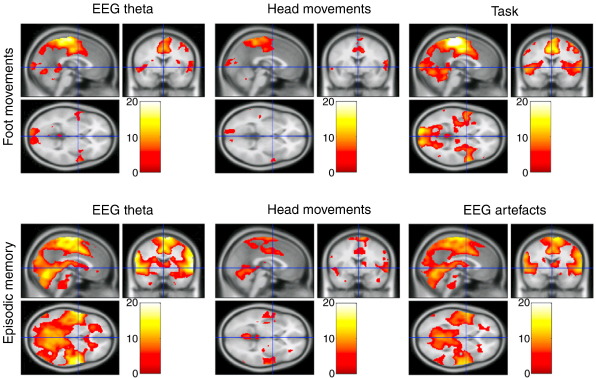
Fixed effects analysis from the foot movement dataset (n = 4; top row) and the episodic memory dataset (n = 4; bottom row) for the convolved models. T2*-weighted signal correlates of cHRF-convolved EEG theta shown in the left column, cHRF-convolved head movements in the middle column, cHRF-convolved foot movement boxcar in the upper right corner and cHRF-convolved visually detected artefacts in the EEG in lower right corner. Fixed effects analysis, threshold at p < 0.05 family-wise error (FWE) corrected. See Table 1 for more details on cluster location and t-scores.

Using convolved regressors to predict the T2*-weighted signal variation for the foot-movement task, a common pattern of results was found for the basic boxcar model characterising periods of foot movement, regressors of head movements and of continuous theta fluctuations models (Analyses 1–3). All exhibited significant positive correlations with predominantly bilateral activity in the mid-cingulate cortex, medial frontal gyrus, precentral gyrus, inferior parietal lobule, lingual gyrus and postcentral gyrus (see Table 1B and Fig. 4). Refer to Supplemental Fig. 2 for individual participant GLM results of the foot movement dataset. The highest inter-participant consistency was found for the basic boxcar and continuous theta predictors.

**Fig. 5:**
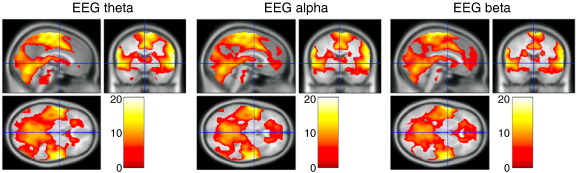
T2* weighted signal correlates of cHRF-convolved EEG theta (left), alpha (middle) and beta (right), for the episodic memory task (n = 4). Fixed effects analysis, threshold at p < 0.05 family-wise error (FWE) corrected.

For the episodic memory fMRI dataset, the analyses of the convolved EEG theta, alpha and beta bands, as well as head movements and visually detected EEG artefacts all resulted in a similar pattern of results, though slightly more widespread as compared with the analyses of the foot-movement task. Positive correlates were found in the mid-cingulate, medial frontal gyrus, cerebellum, precentral gyrus, inferior parietal lobule, insula, thalamus, lingual gyrus, postcentral gyrus, in most cases bilaterally (see Table 1B, Figs. 4 and 5). See Supplemental Fig. 3 for individual participant GLM results of the episodic memory dataset for the predictors formed from EEG theta, head movements and visually detected EEG artefacts. The highest inter-participant consistency was found for the EEG theta and visually detected EEG artefact predictors, as indicated by the t-scores.

## Discussion

Using two different EEG/fMRI datasets, we investigated confounding correlations between motion-related EEG artefacts and fMRI signal intensity: the first, involving a foot movement task which was designed to induce head movements with similar amplitude to those normally found in fMRI experiments in a reasonably predictable manner and the second, based on an episodic memory task, that generated less predictable head movements more typical of standard EEG/fMRI experiments. We estimated the degree of head movements and artefacts using various analysis methods based on the times of cued movement, the fMRI realignment parameters and visually detected large amplitude EEG artefacts. Each method revealed a strikingly similar pattern of correlations between fMRI signal intensity and head movement artefacts in the EEG data when the EEG regressor was convolved with the canonical haemodynamic response function. No consistent pattern of significant correlation was observed in the fMRI data when the regressors were not correlated with the cHRF.

Since motion artefacts are expected to be similar in form in EEG and EMG recordings, some insight that is relevant to this study can be gained by considering the work of van der Meer et al. (2010) who studied motion artefacts in EMG recordings taken from the arm inside an MR scanner. They found that motion artefacts in the EMG signal displayed a pan-spectral distribution up to 30 Hz and exhibited amplitudes as high as 30 mV. This is 20–40 times smaller than muscle activity in EMG recordings (van der Meer et al., 2010), but much larger than the effect of neuronal activity measured in scalp EEG. Our observation of spikes coincident in time in the EEG theta, alpha and beta regressors suggests that movement-related noise spikes uniformly affect wide-ranging frequency bands (Supplemental Fig. 1), agreeing with the findings of van der Meer et al. (2010).

Crucially, the motion-related artefacts in the EEG data only significantly correlated with a large number of brain regions when convolved with the cHRF. Without convolution with the cHRF, no significant correlates of theta fluctuations, abrupt movements or EEG artefacts were found (see Table 1 and Fig. 3). If the EEG regressors had correlated with the T2*-weighted signal without convolution with the cHRF, then it would be likely that movement-related MR signal changes underlie the measured correlations. In this case, one would also predict that the correlations would mainly occur near boundaries between regions of low and high signal intensity (for example, at the surface of the brain) where movement related signal changes are expected to be largest. This spatial pattern was not evident in the correlation map, indicating that direct effects of movement on the MR signal were not the dominant source of correlation. Given that significant correlation only emerged after convolution of regressors with the cHRF, and that the highlighted regions are linked to motor function, these data suggest that the artefacts in EEG data are correlated with neural activity related to head movements in the scanner. In other words, by delaying our EEG artefact predictors in the fMRI GLM model to allow for the slow haemodynamic response, we have detected cortical networks active during small head movements, as reflected in the BOLD signal. The identification of this activation even after the use of stringent methods to remove artefacts in the EEG data suggests that there may be low level residual artefacts that are difficult to identify and remove, which dominate the continuous EEG regressors.

Across studies in the literature there is a large variation in the observed correlations of the different frequency bands with the T2* weighted signals (de Munck et al., 2009; Goldman et al., 2002; Laufs et al., 2003; Scheeringa et al., 2008), it is plausible that these differences originate either in variations in the amount of participant movement or the methods employed for discarding sections of noisy data. One difference between the data included in the current study and those previously reported is that the participants were performing a task (either foot movements or an episodic memory task) in our study and therefore might be expected to undergo more significant head movement than participants who have been instructed to lie still and close their eyes (or keep them open) as in previously reported studies (de Munck et al., 2009; Goldman et al., 2002; Laufs et al., 2003; Moosmann et al., 2003; Scheeringa et al., 2008). It has become clear in this study that it is small movements and those of short durations which are not always picked up by realignment parameters that cause EEG artefacts. Overall however, the realignment parameters in our study indicated that rotations did not exceed 1.7° over the whole time course, which indicates that in this study the magnitude of movements was within the limits regarded as acceptable in many fMRI studies. Furthermore, several of the regions found in this study overlapped with those found by De Munck et al. (2009), which incidentally also demonstrates colinearity between multiple frequency bands.

The pattern of activity in the bilateral posterior cingulate, insula, precentral gyrus, inferior parietal lobule, medial frontal gyrus and thalamus which we identified by correlation to the convolved movement and EEG predictors is consistent with that expected for self-generated movements or processes concomitant with these movements (Toro et al., 2008). BOLD signals in the pre/postcentral gyrus, superior temporal gyrus, middle frontal gyrus, and cingulate gyrus correlate with motor preparation during a motor attention task (Rushworth et al., 2001). Attentional processes, including not only the startle reflex, but also orienting processes, are accompanied by pericranial muscle activation (van Boxtel et al., 1996). Our observed correlations between indicators of head movement and neural activation in attentional networks, is therefore consistent with what we know of attentional processes. We also observed additional active clusters in the lingual gyrus often extended into multiple regions of the visual cortex.

Correlation of theta, alpha and beta regressors formed by convolution with the canonical haemodynamic response function (cHRF) with the T2*-weighted signal, revealed a similar spatial pattern of activity for the three different frequency bands. This pattern of activation corresponds to a network of cortical areas, which has been associated with motor movement (Toro et al., 2008), including the bilateral mid-cingulate, medial frontal cortex, superior temporal gyrus, insula, precentral gyrus, inferior parietal lobule and thalamus (see Figs. 4 and 5). These results suggest that continuous predictors of the T2*-weighted signal created by EEG oscillations contain motion-related noise even after stringent cleaning of the EEG data.

The movement-induced artefacts in the EEG time-course mapped relatively well onto MR realignment parameters seen during the foot movements in some participants (see Fig. 2), however some variance persisted. The between-subject variance became more evident in the analyses where motion predictors, constructed from the derivative of the realignment parameters, were used to predict the T2*-weighted signal variation (see Table 1 and Supplemental Figs. 2 and 3). This method of estimating head movement may be sub-optimal for estimating motion-related EEG artefacts in the scanner since MR realignment parameters give a measure of head movement per volume (2.5 to 3 s in this study). Head movements that generate artefacts in the EEG occur on a much shorter time-scale, and will therefore often not be manifested in the measured realignment parameters. This may have meant that for several participants the movement onsets were not well detected. Methods that detect movement at a higher temporal resolution may improve cleaning techniques for such artefacts, and in turn could enhance the use of EEG data in predicting the T2* weighted signal variation due to the task of interest. Ideally, tracking head movements while a participant is in the MR scanner at a higher temporal resolution than afforded by the realignment parameters would provide a more accurate measurement of onsets and offsets of movements and facilitate potential head-movement template construction. Zaitsev et al. (2006) employed a motion-tracking device to improve realignment of fMRI images. It is possible that a similar approach could be usefully implemented for head movement detection in EEG/fMRI, and this should be investigated in future studies.

Movement artefacts are expected to occur when a movement produces a change in the magnetic flux linked by the conducting loop formed by an electrode lead, the conducting material of the head, and the reference lead. In a uniform magnetic field, pure translation does not produce a change in the flux linkage. However, rotations around the left to right axis (pitch) and the anterior–posterior axis (roll) do change the linked flux. Yan et al. (2010) demonstrated that if it is assumed that the electrode leads follow lines of longitude over the scalp surface, the magnitude of the pitch artefact is linearly proportional to the product of angular velocity and the cosine of the angular displacement, so that the effect varies smoothly with variation in speed and magnitude of the movement. For example, a ‘nod’ movement with the head (i.e. rotating the head forward or backward, chin toward or away from the chest) produces a bipolar scalp topography (Yan et al., 2010). The moderate success of template based methods for removing the pulse-artefact suggests that similar methods might be used to remove artefacts produced by nodding movements unrelated to heart beat, provided the time of occurrence of these movements can be identified. One could theoretically construct a template of movement-related artefacts in the EEG by calculating the bipolar derivatives of the bilateral temporal channels (e.g. T8-T7) to detect ‘nods’, and then fitting the predicted artefact to the observed data, with an adjustable parameter to reflect the speed and amplitude of the movement. Similarly, the bipolar derivative of frontal-parietal channels (e.g. Fz-Pz) might be used to identify roll movement. Exploration of the topography of artefacts found in the data recorded in this study revealed that some artefacts exhibited moderate consistency with the topography predicted by Yan et al. (2010) justifying further investigation of the feasibility of developing a procedure of this type.

In conclusion, the current findings suggest that head movements confound inferences drawn from the integration of simultaneously acquired EEG and fMRI data, and that the effect of head movements may dominate even after the EEG signal has been ‘cleaned up’. The effect of such artefacts is potentially magnified in analyses using continuous EEG predictors of the fMRI signal. In studies where the analysis does not depend on the full time-span of recorded EEG data, it is possible to select only data segments that are unaffected by artefacts. However, because movements are particularly likely around the times of greatest interest, such as when responding to stimuli, developing an appropriate technique to detect and remove these is more likely to yield the preferred solution.

The following are the supplementary materials related to this article.

Supplemental Fig. 1 Time course in fMRI volumes of theta (blue), alpha (red) and beta (green) EEG signals that were convolved with cHRF and modelled as regressors in the GLM analyses, for one representative participant during the first session of the episodic memory task. The time courses display strong inter-correlations (all r > 0.8, all p < 0.0001).

Supplemental Fig. 2 FMRI analysis of the foot movement task data. FMRI signal correlates of EEG theta amplitude (first column), onsets of movements derived by realignment parameters (middle column) and task (last column) shown for the four participants (S1–S4) during the foot movement task. Results are displayed at a threshold of p = 0.001 uncorrected.

Supplemental Fig. 3 fMRI analyses of the episodic memory task data. FMRI signal correlates of EEG theta amplitude (first column), onsets of movements derived by the realigment parameters (middle column) and onsets of visually detected noise in the EEG (last column) shown for the four participants (S1– S4) during the episodic memory task. Results are displayed at a threshold of p = 0.001 uncorrected.

## Figures and Tables

**Table 1:** Significant peak voxel coordinates in EEG/fMRI fixed-effect analyses: A) regressors not convolved with the cHRF, and B) regressors convolved with the cHRF. Region names were derived using the WFU PickAtlas toolbox plugin for SPM (http://fmri.wfubmc.edu/software/PickAtlas). Locations of peak T-value voxels are given in MNI coordinates with their associated T-value, all significant at p < 0.05 corrected for family-wise error rate. Only clusters of more than 20 voxels are reported.

	Dataset 1: foot movements						Dataset 2: episodic memory					
	EEG theta (4–8 Hz)		Head movements		Task		EEG theta (4–8 Hz)		Head movements		EEG artefacts	
	[*x y z*]	*t*	[*x y z*]	*t*	[*x y z*]	*t*	[*x y z*]	*t*	[*x y z*]	*t*	[*x y z*]	*t*
*A: Region name*			[12 − 12 18]	5.5								
Thalamus R			[34 − 50 − 12]	5.5								
Fusiform gyrus R			[26 − 72 − 4]	5.3								
Lingual gyrus R			[40 − 66 − 8]	5								
Middle occipital gyrus R											[34 34 14]	6.3
Inferior frontal gyrus R												


*B: Region name*												
Mid-cingulate R	[10 − 14 42]	12.4	[12 − 14 42]	7.8	[8 2 46]	14.49	[6 8 44]	15.2	[2 − 8 44]	7.1	[10 6 42]	11.7
Mid-cingulate L	[− 8 − 14 40]	13.4	[− 6 − 16 42]	9.3	[− 10 − 14 40]	18.95	[− 8 − 8 46]	13.9	[− 6 − 8 40]	6.6	[− 6 10 38]	10.6
Medial frontal gyrus R	[2 − 20 68]	18.5	[4 − 24 70]	9.9	[2 − 20 68]	26.7	[6 − 14 56]	15.5			[6 − 10 56]	11.4
Medial frontal gyrus L							[− 8 − 12 56]	13.1				
Cerebellum L	[− 4 − 74 − 12]	6.6			[− 6 − 74 − 14]	10	[− 2 − 72 − 16]	14.9	[− 8 − 70 − 14]	8.2	[− 6 − 68 − 14]	12.4
Superior temporal gyrus R	[62 4 − 4]	7.6	[62 2 − 2]	5.9	[60 4 − 2]	11.8	[62 − 14 6]	17.5	[62 2 − 6]	7.2	[60 0 − 2]	12.6
Superior temporal gyrus L	[− 58 6 − 10]	8			[− 58 6 − 10]	12.2	[− 60 4 − 2]	16.7	[− 54 6 − 6]	6	[− 56 8 − 6]	12.7
Inferior parietal lobule R	[56 − 38 28]	9.6	[62 − 42 28]	6.3	[54 − 36 26]	13.2	[54 − 38 22]	14.4				
Inferior parietal lobule L							[− 48 − 38 24]	15.1	[− 46 − 38 24]	6.8	[− 44 − 38 22]	13.3
Insula R	[34 − 26 14]	7.8			[44 − 26 14]	13.4	[30 − 28 16]	16.1			[40 − 36 18]	10.7
Insula L	[− 50 − 30 18]	9			[− 34 − 24 6]	10.5	[− 32 − 16 14]	15.6	[− 34 − 14 6]	4.9	[− 34 − 14 10]	9.9
Thalamus R					[16 − 18 10]	6.6	[18 − 24 12]	10.8	[10 − 4 14]	5.4	[6 − 4 10]	8.3
Thalamus L	[− 18 − 20 14]	5.7			[− 20 − 22 10]	6.8	[− 20 − 24 14]	10.5			[− 18 − 26 18]	8.4
Lingual gyrus R	[14 − 100 0]	6.1	[10 − 88 − 6]	5.2	[14 − 100 0]	12.5	[20 − 68 4]	12.2			[22 − 64 4]	7.8
Lingual gyrus R	[− 8 − 102 4]	8	[− 12 − 88 − 2]	5.2	[− 14 − 98 − 2]	15	[− 14 − 70 2]	11.8			[− 16 − 70 2]	7.4
Postcentral gyrus R	[8 − 40 74]	19.5			[64 − 26 16]	12	[20 − 32 62]	18.6	[26 − 28 74]	10.4	[22 − 30 74]	14.8
Postcentral gyrus L	[− 6 − 44 74]	17.6			[− 50 − 28 16]	13.1	[− 18 − 34 72]	18.8	[− 48 − 16 10]	7	[− 18 − 30 74]	16.2
Superior temporal gyrus R	[56 10 − 10]	8.3	[66 − 32 14]	7.8	[64 − 30 16]	13.2	[60 − 34 18]	14.7	[60 − 36 18]	7.3	[60 − 32 16]	12.1
Superior temporal gyrus L			[− 60 − 34 14]	6.6	[− 34 − 24 8]	10.9					[− 50 − 26 8]	10.9
Middle frontal gyrus R							[36 46 22]	9.8	[30 40 22]	6.8	[34 46 26]	7.3
Middle frontal gyrus L							[− 30 38 28]	8.6			[− 28 38 30]	7.2
Precentral gyrus R							[− 58 − 4 30]	18.3	[52 − 10 24]	6.7	[58 − 2 32]	11.8
Precentral gyrus L							[52 − 10 28]	17.3	[− 60 − 8 18]	7	[− 60 − 10 28]	13.1
Superior frontal gyrus R							[12 − 6 76]	20.5	[8 − 8 74]	11.5	[8 − 6 74]	16.5
