# Supplementary figures and images for: Motion-related artefacts in EEG predict neuronally plausible patterns of activation in fMRI data
Source: Neuroimage. 2012 Jan 2;59(1-3):261–70. doi: 10.1016/j.neuroimage.2011.06.094 (PMC3221044; doi:10.1016/j.neuroimage.2011.06.094)

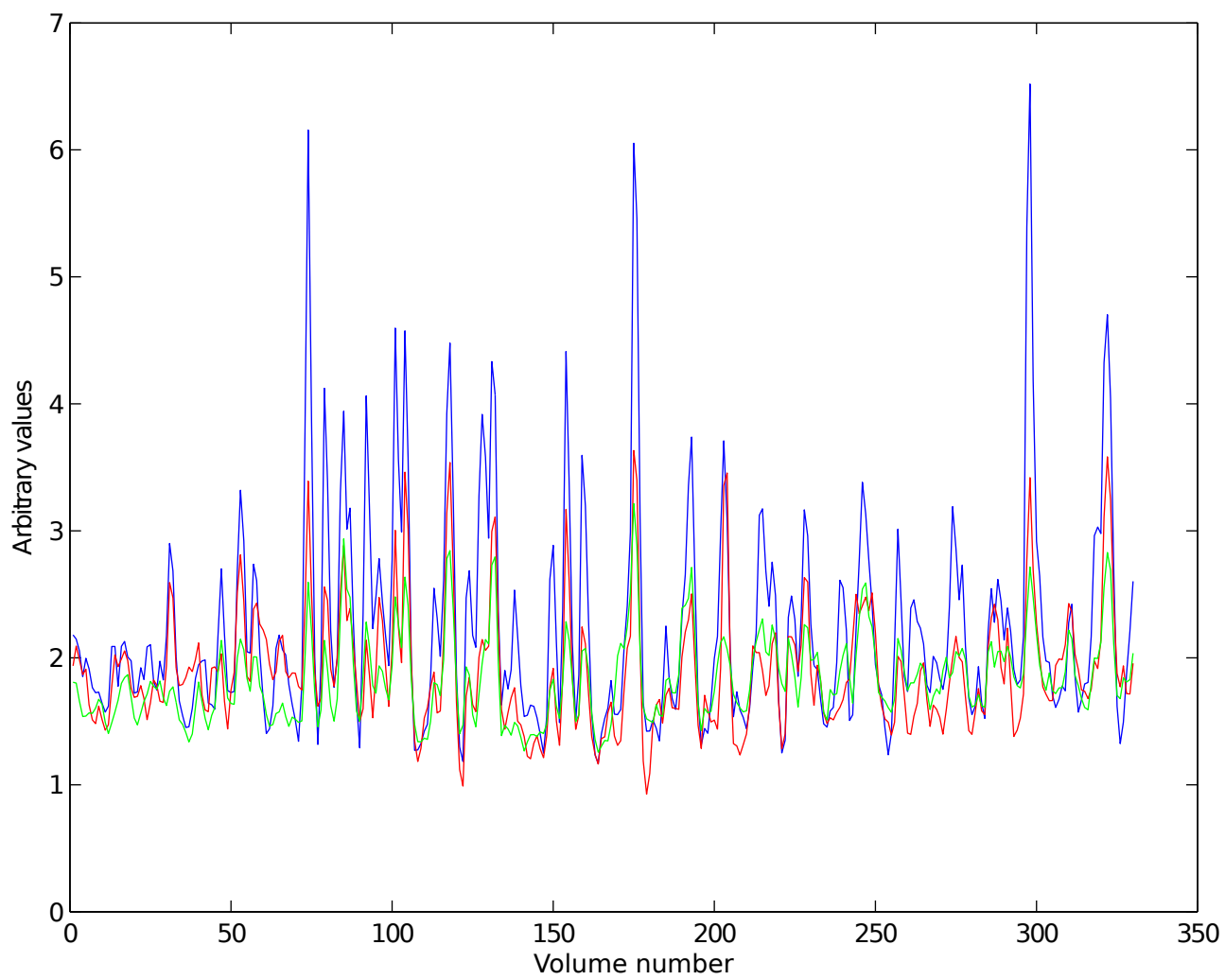

Supplement: Supplemental Fig. 1 — Time course in fMRI volumes of theta (blue), alpha (red) and beta (green) EEG signals that were convolved with cHRF and modelled as regressors in the GLM analyses, for one representative participant during the first session of the episodic memory task. The time courses display strong inter-correlations (all r > 0.8, all p < 0.0001). [file mmc1.pdf]

### Correlates of “theta”

### Correlates of “movements”

### Correlates of “task”

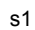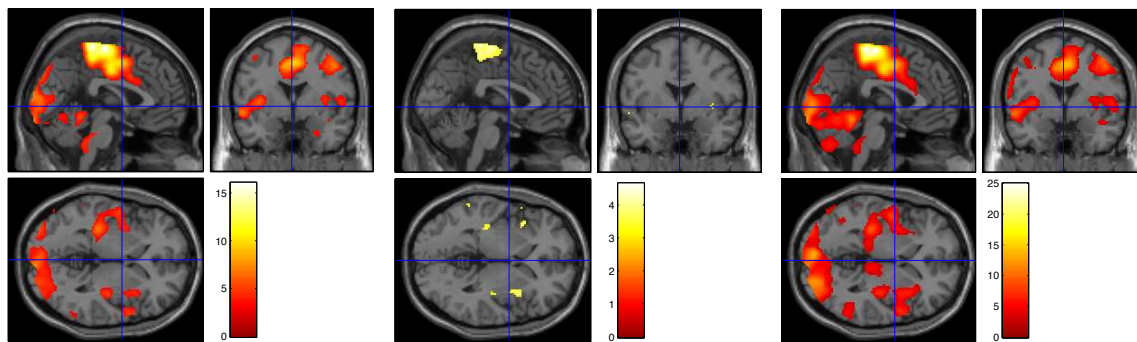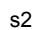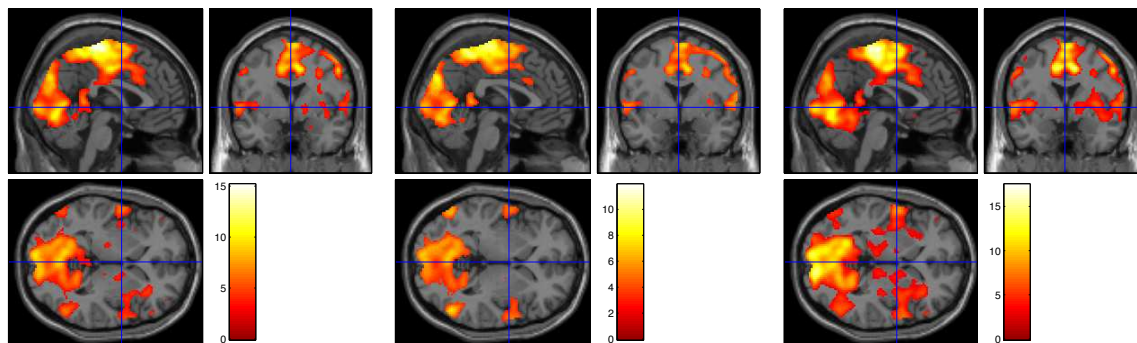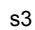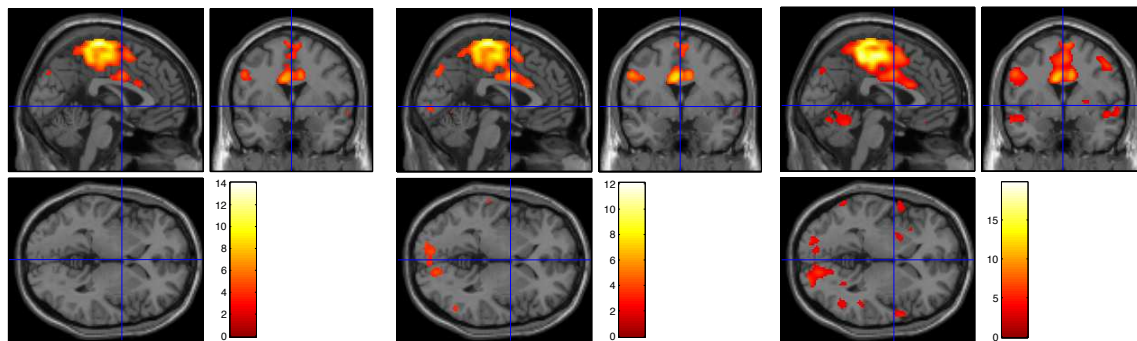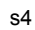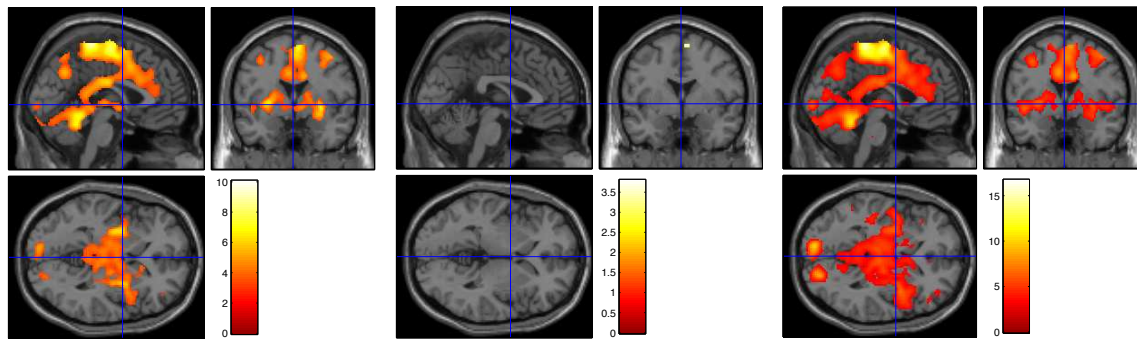

Supplement: Supplemental Fig. 2 — FMRI analysis of the foot movement task data. FMRI signal correlates of EEG theta amplitude (first column), onsets of movements derived by realignment parameters (middle column) and task (last column) shown for the four participants (S1–S4) during the foot movement task. Results are displayed at a threshold of p = 0.001 uncorrected. [file mmc2.pdf]

Correlates of "theta"

Correlates of "movements"

Correlates of "EEG noise"

s1

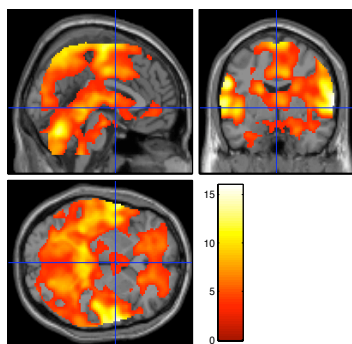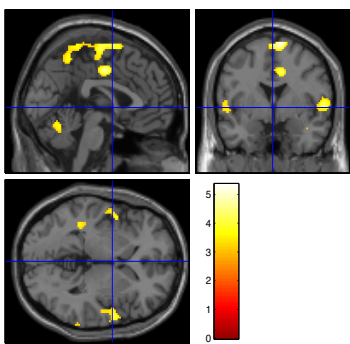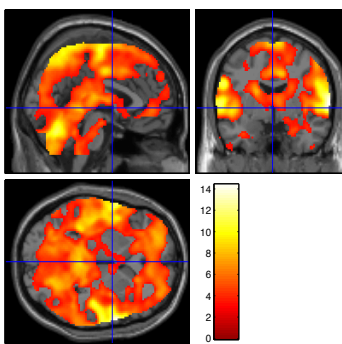

s2

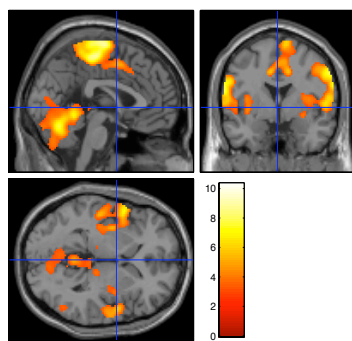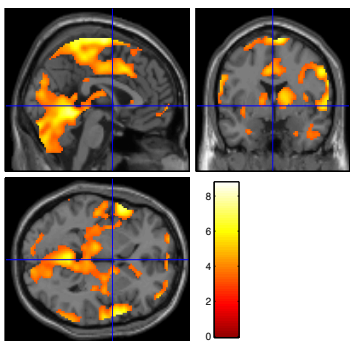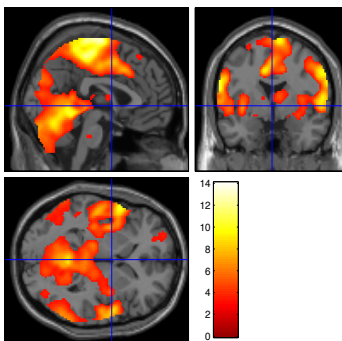

s3

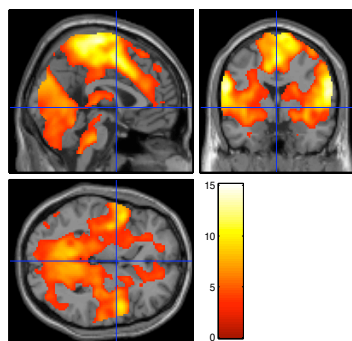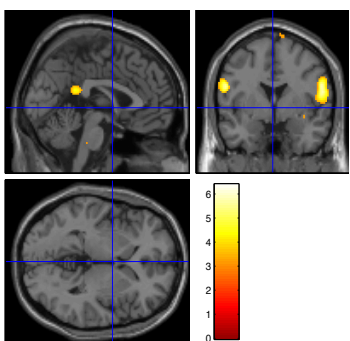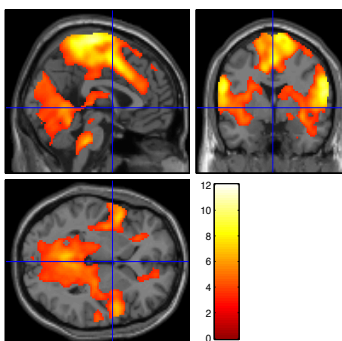

s4

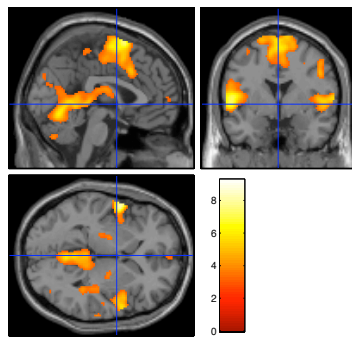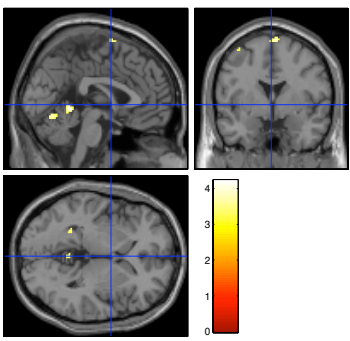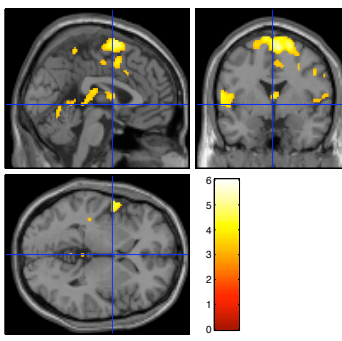

Supplement: Supplemental Fig. 3 — fMRI analyses of the episodic memory task data. FMRI signal correlates of EEG theta amplitude (first column), onsets of movements derived by the realigment parameters (middle column) and onsets of visually detected noise in the EEG (last column) shown for the four participants (S1– S4) during the episodic memory task. Results are displayed at a threshold of p = 0.001 uncorrected. [file mmc3.pdf]
